# A Large Rice Body-Containing Cyst Mimicking Infection following Total Hip Arthroplasty: A Case Report

**DOI:** 10.1155/2017/5354298

**Published:** 2017-06-05

**Authors:** Wael Bayoud, Maroun Rizkallah, Samuel Georges, Tonine Younan, Gaby Haykal

**Affiliations:** ^1^Orthopedic Surgery Department, Hotel-Dieu de France Hospital, Achrafieh, Beirut, Lebanon; ^2^Radiology Department, Hotel-Dieu de France Hospital, Achrafieh, Beirut, Lebanon

## Abstract

**Introduction:**

Soft tissue mass following total hip arthroplasty raises several differential diagnoses not limited to infection, hematoma, wear debris, malignancy, and bursitis. Rice body formation in the hip region is an uncommon process denoting a chronic inflammation. We report here the second case of its kind in the medical literature of a wide symptomatic rice-like body cyst complicating a total hip arthroplasty.

**Case Presentation:**

This is the case of an 82-year-old white female, presenting with a warm, red, and inflated groin five years after revision of right total hip arthroplasty. Surgical intervention reveals a large well circumscribed cyst containing well-organized rice-like bodies. This eventuality was never reported in differential diagnosis of hip periprosthetic soft tissue masses before.

**Conclusion:**

This case report helps widening the array of the differential diagnosis in patients presenting with a slow growing soft tissue mass following total hip arthroplasty, making rice-like bodies cyst a valid one to consider.

## 1. Introduction

There is a wide array of possible differential diagnosis in front of an organized soft tissue mass following total hip arthroplasty [1]. These include infection, hematoma, wear debris, instability, malignancy, synovial cysts, and bursitis [2]. Characteristic findings on physical exam as well as radiologic and serologic examinations can make the diagnosis straightforward in the majority of cases [1]. However, some etiologies are rare and accurate diagnosis waits for percutaneous drainage or even surgical intervention. We present here the case of an 82-year-old female patient who developed a slowly growing nontender inflammatory large painful hip mass 5 years following a revision total hip arthroplasty.

## 2. Case Presentation

An 82-year-old female patient presented to our clinics following progressive right hip nontender erythema, warmth, and swelling, with no functional impotence worsening progressively during the last month. She has no fever and no general weakness or loss of weight or any sign of infection or systemic disease.

She has a past medical history significant for hypertension and well controlled diabetes with bilateral total hip arthroplasty for osteoarthritis. On the left asymptomatic side, she underwent a total hip arthroplasty at the age of 60 which was complicated, at age of 78, of periprosthetic infection and was treated with a two-stage revision of total hip arthroplasty. On the right symptomatic side, she underwent total hip arthroplasty at age of 67 which was complicated of nonunion of greater trochanter treated surgically with wiring at age of 74 and posterior dislocation at age of 77 that was treated surgically with a one-stage revision of the acetabulum due to instability. She was completely asymptomatic back then until the last month when she noted erythema, warmth, and swelling of the right hip. There was no history of rheumatoid arthritis, general systemic disease, or chronic infection conditions such as tuberculosis.

**Figure 1:**
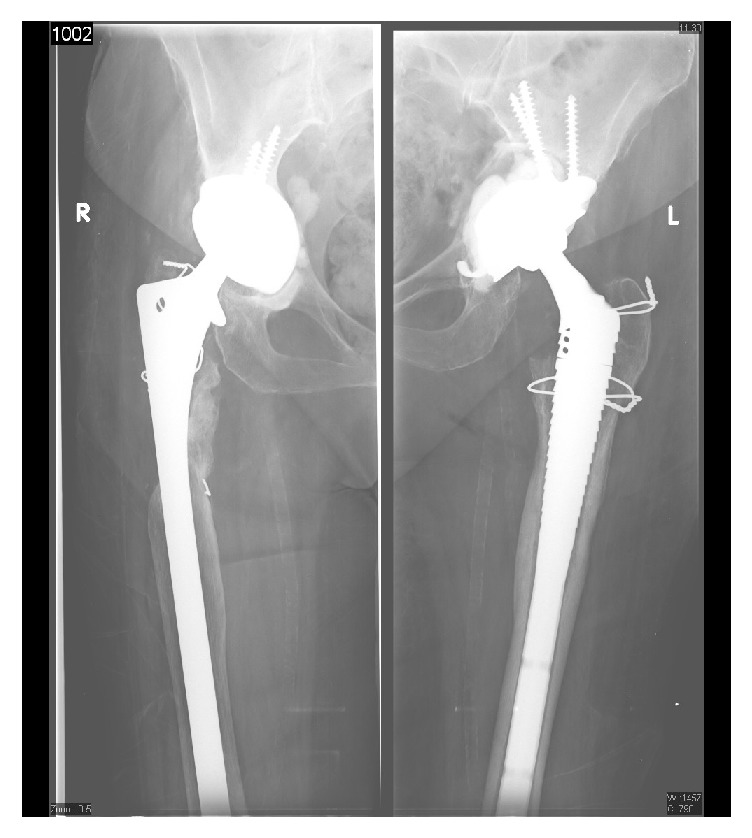
Preoperative A/P left and right hip X-rays of the patient. This figure shows an AP radiograph of the left and right hips of the patient in the immediate preoperative setting.

**Figure 2:**
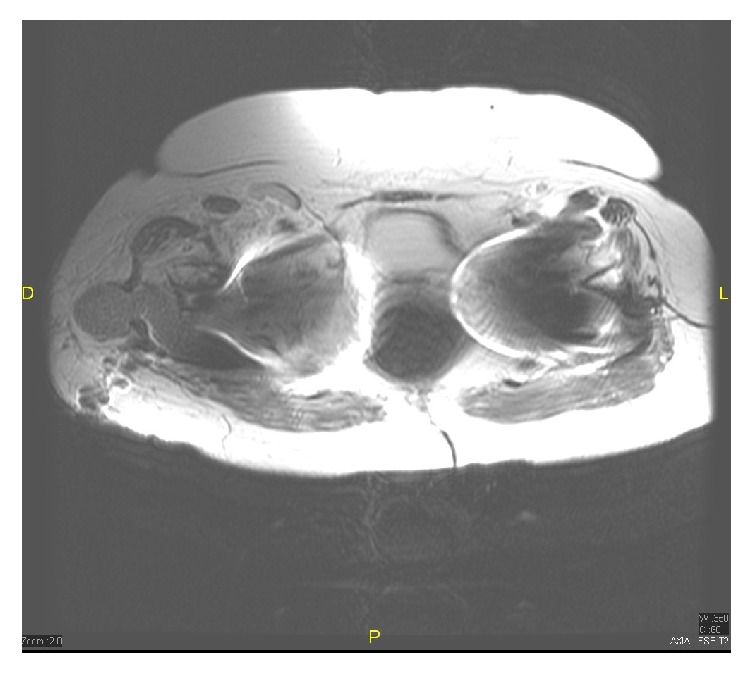
A preoperative right hip MRI. This is a T2 fat-sat weighted MRI axial cut showing the well-organized collection extending into the right prosthesis.

Laboratory investigations were unremarkable with a normal WBC count, except for a CRP of 10 ng/mL and an ESR of 45 mm/hr. Radiographs of the pelvis showed bilateral noncemented total hip arthroplasty (metal on polyethylene) with no significant soft tissue changes (Figure 1). No cyst or mass was visualized. A magnetic resonance imaging (MRI) of the pelvis demonstrated a large well-circumscribed hematoma collection with inflammatory changes and cystic areas facing superficially the right hip prosthetic material but not surrounding it with an intermediate signal on T1- and T2-weighted images (Figure 2). The yield of this imaging technique was extremely limited by the artifacts coming from the prosthesis. A tentative of percutaneous puncture under X-ray guidance failed to yield any liquid.

**Figure 3:**
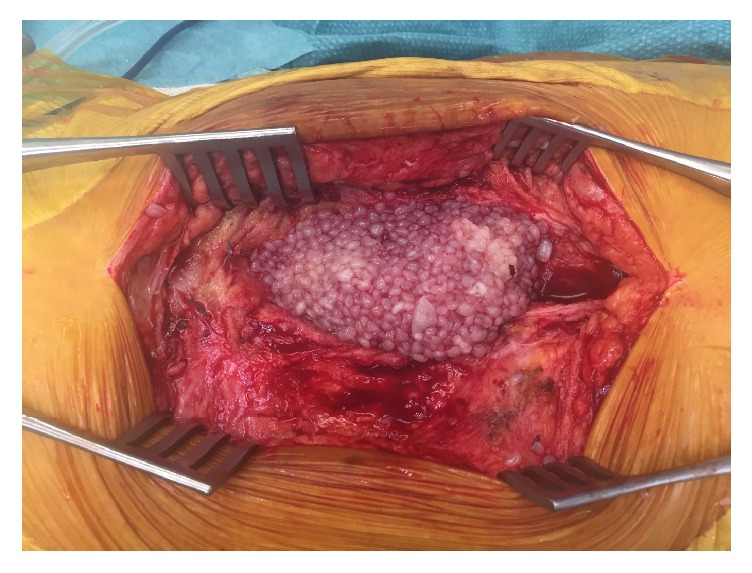
A per-operative view of the rice-like body cyst. The rice-like body cyst discovered in the per-operative setting. The rice-like bodies are seen bathing in a clear fluid. An encapsulated cyst.

Surgical exploration was performed through an excision of the old lateral wound and by adopting the classic lateral approach of the hip. After cutting the gluteus medius, a large cystic mass formation was noted, overlying the hip joint and extending into it. This well contoured mass is opened revealing a collection of 250 mL of “rice-like” bodies measuring 1 cm each bathing in a clear fluid (Figure 3). It was completely excised and sent to culture and anatomopathological studies. Cultures were sent to look for aerobic, anaerobic, fungal, and acid fast bacilli. There was no evidence of necrotizing or infected or malignant tissue. The hip prosthesis was in place with no evidence of loosening. The wound was copiously irrigated and closed in layers using absorbable suture over drain and stapler on the skin.

**Figure 4:**
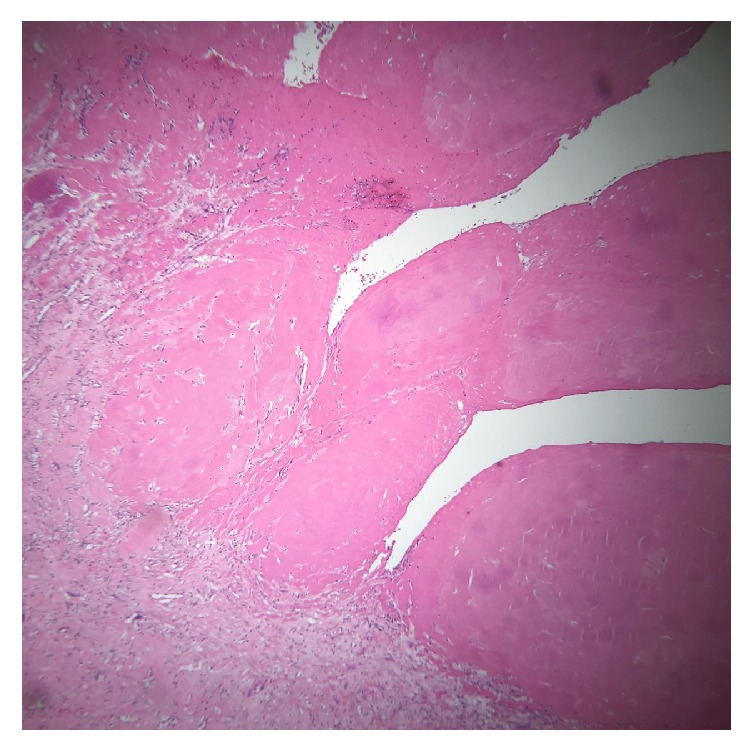
Anatomopathological view of the operative material. Anatomopathological view of the material sent to pathology showing a fibrotic material with a cystic wall with fibrinous projections in the cystic cavity compatible with rice-like bodies.

All the taken cultures came back negative. The PPD was nonreactive. The RF and the anti-CCP serology were also negative. The microscopic study of the cystic collection and its content revealed a fibrotic synovial nature of cystic wall with fibrinous projections in the cystic cavity similar to the foreign objects qualified as “rice-like” bodies (Figure 4). In fact, they appeared to be fibrinous formation with a collagenous fibrosis. There was no evidence of granulomatosis formation or amyloid deposition. The patient had an uncomplicated postoperative course, the wound healed, and she was asymptomatic at two months of follow-up.

## 3. Discussion

These formations are very rare and usually asymptomatic in the deep hip joint. However, they may cause pain by compressing nearby structures and groin edema when located anterolaterally [1, 3]. Inflammatory signs and symptoms (redness, warmth), mimicking infection, are present when the mass is growing in size. In the present case, we acted to rule out infection, especially with the patient having a history of contralateral periprosthetic joint infection. Serologic inflammatory markers showed borderline values but the preserved general status of the patient was against this eventuality. MRI findings could not rule out infection. A percutaneous X-ray guided puncture was tried but it failed to yield any fluid. This is probably because of the fibrinous nature of the rice bodies that blocked the needle tip upon negative pressure instauration. Therefore, no preoperative culture was possible. In the immediate preoperative setting, we were left with the following differential diagnosis: infection with organized abscess, organized hematoma, chronic bursitis, pigmented villonodular synovitis, synovial osteochondromatosis, and malignancy.

MRI is considered as the imaging modality of choice for rice bodies which should appear as isointense or hypointense bodies in a magma of hyper-intense liquid on T2-weighted images [4–6]. Some imaging features may help distinguish rice bodies from pigmented villonodular synovitis and synovial osteochondromatosis [5, 6], which are the main two differential diagnoses of this condition. But, MRI was not of a value in this specific case because of the artifact coming from the metallic prosthesis.

In front of a suffering patient, observation and conservative therapy are not an option especially that an active infection could not be ruled out. Percutaneous puncture and drainage which was indicated in this case according to the clinical and imaging findings failed [1]. We opted for complete surgical excision and drainage with culture and anatomopatologic studies that yielded the diagnosis of a large, isolated, highly organized, periprosthetic rice-like bodies cyst.

This is the second case of periprosthetic hip joint rice-like bodies cyst to be reported in literature [2]. This eventuality was never reported in differential diagnosis of hip periprosthetic soft tissue masses before [7–10]. Therefore, our case report, together with the previous case published in 2012, is adamant in widening the array of the differential diagnosis in patients presenting with a slow growing soft tissue mass following total hip arthroplasty, making rice-like bodies cyst a valid one to consider.

## 4. Conclusion

Despite many differentials that should be aroused in front of a slow growing symptomatic soft tissue mass complicating total hip replacement, a usually uncommon but actually more and more encountered possibility should be considered. This is the slow growing rice-like body cyst that may be found outside systemic conditions as a witness of a chronic local inflammatory condition. Therefore, as a result of our case report and of the one previously published, physicians should keep in mind this relatively uncommon condition when dealing with patients having a similar presentation.

## References

[1] Yukata K., Nakai S., Goto T. (2015). Cystic lesion around the hip joint. *World Journal of Orthopaedics*.

[2] Issack P. S. (2012). Formation of a large rice body-containing cyst following total hip arthroplasty. *BMC Research Notes*.

[3] Forse C. L., Mucha B. L., Santos M. L. Z., Ongcapin E. H. (2012). Rice body formation without rheumatic disease or tuberculosis infection: A case report and literature review. *Clinical Rheumatology*.

[4] Spence L. D., Adams J., Gibbons D., Mason M. D., Eustace S. (1998). Rice body formation in bicipito-radial bursitis: Ultrasound, CT, and MRI findings. *Skeletal Radiology*.

[5] Griffith J., Peh W., Evans N., Smallman L., Wong R., Thomas A. (1996). Multiple rice body formation in chronic subacromial/subdeltoid bursitis: MR appearances. *Clinical Radiology*.

[6] Chen A., Wong L.-Y., Sheu C.-Y., Chen B.-F. (2002). Distinguishing multiple rice body formation in chronic subacromial-subdeltoid bursitis from synovial chondromatosis. *Skeletal Radiology*.

[7] Potter H. G., Nestor B. J., Sofka C. M., Ho S. T., Peters L. E., Salvati E. A. (2004). Magnetic resonance imaging after total hip arthroplasty: Evaluation of periprosthetic soft tissue. *Journal of Bone and Joint Surgery - Series A*.

[8] He C., Lu Y., Jiang M., Feng J., Wang Y., Liu Z. (2014). Clinical value of optimized magnetic resonance imaging for evaluation of patients with painful hip arthroplasty. *Chin. Med. J. (Engl*.

[9] Hayter C. L., Koff M. F., Potter H. G. (2012). Magnetic resonance imaging of the postoperative hip. *Journal of Magnetic Resonance Imaging*.

[10] Fritz J., Lurie B., Miller T. T. (2013). Imaging of hip arthroplasty. *Seminars in Musculoskeletal Radiology*.

